# Editorial: Probiotic Trigger Molecules in Action

**DOI:** 10.3389/fmicb.2021.789209

**Published:** 2021-11-25

**Authors:** Jasna Novak, Emmanuelle Maguin, Afef Najjari, Konstantinos Papadimitriou

**Affiliations:** ^1^Faculty of Food Technology and Biotechnology, University of Zagreb, Zagreb, Croatia; ^2^MIHA Laboratory, UMR MICALIS, Université Paris Saclay, Institut National de Recherche pour l'Agriculture, l'Alimentation et l'Environnement (INRAE), AgroParisTech, Jouy-en-Josas, France; ^3^Faculté des Sciences de Tunis, Université de Tunis El Manar, LR03ES03 Microorganismes et Biomolécules Actives, Tunis, Tunisia; ^4^Department of Food Science and Technology, School of Agriculture and Food, University of Peloponnese, Tripoli, Greece

**Keywords:** microbiome, probiotic, post-biotic, trigger molecule, probiotic-host interaction, microbiota

The application of probiotics extends from their importance in food fermentations to the recently recognized therapeutic potential for the alleviation of various disorders through effects on the human microbiome. The study of the latter has been influenced by the growing appreciation of its importance to host's health and the recognition that many disorders may arise from its disruption, a condition called dysbiosis. This is why the Research Topic “*Probiotic trigger molecules in action*” is dedicated to the molecular mechanisms behind probiotic-host interactions, triggered by specific biomolecules. Namely, the biodiversity and functionalities of probiotics and their biomolecules which trigger the observed health benefit are enormous.

In a series of articles presented in this Research Topic, the authors reported the results of characterization of new probiotic candidate strains and proposed strategies to modulate unbalanced microbiome (Ferrer et al.; Rosier et al.). Certain research groups presented results related to the characterization of model probiotic strains. In this context lipoproteins of *Lactobacillus plantarum* WCFS1 were elucidated as critical mediators of immune system recognition, while another team detected for the first time the presence of extracellular acyl-hydrolase of *Lactobacillus rhamnosus* GG and *Bifidobacterium longum* NCC 2705 (Lee et al.; Manasian et al.). Also, several other surface molecules have been proposed as effectors of probiotic activities. The emphasis throughout the topic was on their structural and functional characterization (de Rezende Rodovalho et al.; Lee et al.; Mizuno et al.; Qiao et al.).

Given the biodiversity of probiotic strains and the complexity of the target triggering molecules, accompanied by their commercialization, the challenge is to define the effects of specific compounds. Moreover, there is no clear definition and currently, there are several commonly used terms for probiotic activity triggering molecules, like effectors, biomolecules, post-biotics (Lima Batista et al.). In this study, the authors also comment on parabiotics that represent inactivated microbial cells of probiotics. The molecular palette ranges from widely present products of metabolism, such as lactic acid to macromolecules. In general, those include secreted lower-molecular weight metabolites, antibiotic-like compounds, bioactive peptides such as bacteriocins (Atassi et al.), surface-exposed cell components (such as lipoproteins, S-layer proteins, pili, lipoteichoic acid, etc.) which are the first to interact with the microenvironment or those released into it such as exopolysaccharides (Mizuno et al.) and even enzymatic activities (acyl-hydrolase) or complete structures such as extracellular vesicles (EV) (de Rezende Rodovalho et al.). In this regard, Manasian et al. reported the potential of the lypolytic enzymes *Lr*Lyp from *L. rhamnosus* GG and *Bf* Lyp *B. longum* to affect host lipid metabolism and even other metabolites containing acyl ester bonds in the structure. These proteins exhibited enzymatic activity toward para-nitrophenyl (pNP) esters. In another study, Lee et al. constructed *lgt*-deleted strain, to define the roles of cell lipoproteins in the host-cell interactions of *L. plantarum* WCFS1 using proteomic analysis. Lgt is encoding a prolipoprotein diacylglyceryl transferase, responsible for lipidation of lipoprotein precursors. According to results, *L. plantarum* WCFS1 Δ*lgt* derivative stimulated more pro-inflammatory responses in peripheral blood mononuclear cells (PBMCs) compared to its parental strain. By influencing the host‘s anti-inflammatory responses, lipoproteins can obviously act as important mediators in the recognition of probiotics by the immune system, which is important in the context of inducing tolerance.

Furthermore, challenges and perspectives related to probiotic trigger molecules i.e., post-biotics, but also beyond, including prebiotics, synbiotics, and parabiotics were discussed in two review articles (Daliri et al.; Lima Batista et al.). The first relates to the use of probiotic-based strategies in the treatment of specifically 5-fluorouracil induced intestinal mucositis during chemotherapy. *In vivo* animal or *in vitro* studies of intestinal mucositis revealed the potential of probiotic strains to modulate the immune response and restore intestinal microbiota. Although probiotic-based strategies are promising, their application in immunocompromised patients remains limited since the administration of live bacteria leads to controversial clinical findings. Daliri et al. discuss the molecules which trigger the mechanism underlying specific probiotic activities in the host. These include regulation of the intestinal epithelial barrier function, immunomodulatory capacities, microbiome modulation, modulation of systemic metabolic responses, and recently recognized role in modulation of the gut-brain axis.

Two research articles are devoted to the functional characterization of the exopolysaccharides (EPS) from two lactic acid bacteria (LAB) strains as potential effectors. Notararigo et al. described a specific O2-substituted-(1–3)-β-D-glucan from *Pediococcus parvulus* 2.6. In an *ex vivo* model of Crohn's disease biopsy, treatment with this EPS resulted in an anti-inflammatory effect by reducing the pro-inflammatory cytokine IL-8, both at the level of gene expression and at lowering the concentration. The overall results show that the O2-substituted-(1–3)-β-D-glucan have a potential role in ameliorating inflammation via the gut immune system cell modulation. Moving to antiviral activities, Mizuno et al. presented the potential of the EPS from *Streptococcus thermophilus* ST538 to modulate antiviral immune response triggered by TLR3 activation in porcine intestinal epitheliocytes as revealed by comparative studies of cell-free culture supernatants and fermented skim milk produced by Δ*epsB* and Δ*epsC* mutants.

Most of the probiotic strains are applied to perform health effects at specific sites in gastrointestinal tracat (GIT). In this topic, the probiotic interventions during specific dysbiosis are also explored, to show the biotherapeutic potential of this currently less understood application. Several studies have investigated the therapeutic application of probiotics to modulate gut microbiota but also the impact on the microbiome of the oral cavity and urogenital tract. In the first study, Atassi et al. demonstrated the inhibitory activity of the lactic acid synthesized by vaginal microbiota strains *L. gasseri* and *L. crispatus* against pathogens associated with bacterial vaginosis and urinary tract infections. However, only a limited number of resident *L. gasseri* and *L. crispatus* strains showed the ability to release specific antibiotic-like compounds. Their purification and identification remain challenging due to their low molecular weight and structural instability. In another article, Rosier et al. focused on the screening for new nitrate-reducing *Rothia* strains as probiotic candidates for the treatment of oral cavity related infections. Since these are not GRAS bacteria and since the fact that certain strains can be potentially involved in endocarditis, *Rothia* candidates for oral probiotics must undergo a rigorous safety assessment. Selected oral strains exhibited efficiency to reduce nitrate however clinical studies should test the effects of these treatments on oral and systemic health. Another article from the same research group reports on the topical application of oral probiotic *Streptococcus dentisani* 7746, which improved clinical features associated with oral health. Although the mechanisms underlying the beneficial effect e.g., salivary flow, need to be further elucidated, it is speculated that the antimicrobial activity of S. *dentisani* is related to bacteriocins and to the strains pH-buffering capacity through the arginolytic pathway. Metabolites of another unconventional probiotic candidate, *Paenibacillus bovis* sp. nov. BD3526 originally isolated from Tibetan yak milk, strongly affect the immune capacity of intestinal epithelial cells (Qiao et al.). Single-cell RNA sequencing revealed that BD3526 metabolites of *P. bovis* sp. nov. BD3526 strongly affected the immune capacity of intestinal epithelial cells and mediated the lowering of the abundance of adipocytes in the colon tissue of GK rats.

Finally, this topic also includes the potential, not only of surface-exposed or secreted (macro) molecules but also those packed inside EVs to elicit a beneficial effect on the host. de Rezende Rodovalho and colleagues characterized the EVs of the probiotic dairy bacterium *Propionibacterium freudenreichii* CIRM-BIA 129 and thus exposes immunomodulatory proteins to interact with the host. EVs of this strain entrap a variety of proteins. According to proteomic analysis, some of those include metabolism-related proteins even moonlight proteins, but also proteins involved in cell-host interactions such as immunomodulatory surface-layer protein SlpB. Interactions between EV proteins and human immunomodulatory proteins, and in particular NF-κB1, were predicted *in silico*. This predicted interaction is of functional significance because EVs modulate inflammatory responses by IL-8 release and NF-κB activity in HT-29 human intestinal epithelial cells. This study sets the basis to employ probiotic-derived EVs for the development of bioactive products with therapeutic effects.

In summary, this topic is organized to update the current status of research in the field of probiotic triggering molecules. The collection of articles offers an overview of investigations that assesses current limitations in understanding specific effector molecules or even distinctive structures such as EV. At the same time, the current knowledge in probiotic research reported here, enabled the identification of some yet unexplored functions of probiotics to stimulate future research in the field.

## Author Contributions

JN initiated the Research Topic and invited editors EM, AN, and KP. The editorial was written jointly by the editors of the topic. All authors listed have made a substantial, direct, and intellectual contribution to the work and approved it for publication.

## Conflict of Interest

The authors declare that the research was conducted in the absence of any commercial or financial relationships that could be construed as a potential conflict of interest.

## Publisher's Note

All claims expressed in this article are solely those of the authors and do not necessarily represent those of their affiliated organizations, or those of the publisher, the editors and the reviewers. Any product that may be evaluated in this article, or claim that may be made by its manufacturer, is not guaranteed or endorsed by the publisher.

